# Deciphering Lipid Arrangement in Phosphatidylserine/Phosphatidylcholine Mixed Membranes: Simulations and Experiments

**DOI:** 10.1021/acs.langmuir.3c03061

**Published:** 2023-12-14

**Authors:** Agata Żak, Ksenia Korshunova, Natan Rajtar, Waldemar Kulig, Mariusz Kepczynski

**Affiliations:** † Faculty of Chemistry, Jagiellonian University, Gronostajowa 2, 30-387 Kraków, Poland; ‡ Department of Physics, University of Helsinki, P.O. Box 64, FI-00014 Helsinki, Finland

## Abstract

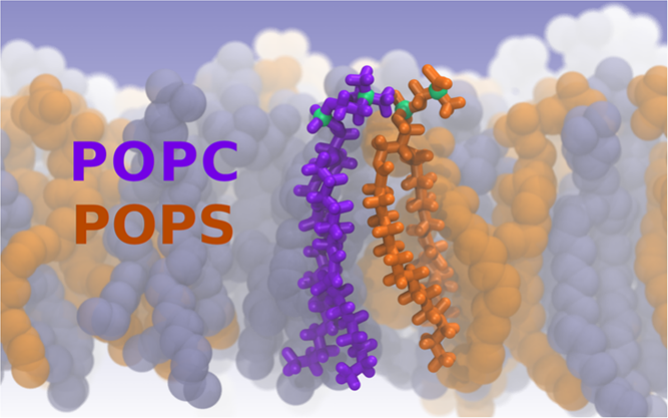

Phosphatidylserine (PS) exposure on the plasma membrane is crucial for many cellular processes including apoptotic cell recognition, blood clotting regulation, cellular signaling, and intercellular interactions. In this study, we investigated the arrangement of PS headgroups in mixed PS/phosphatidylcholine (PC) bilayers, serving as a simplified model of the outer leaflets of mammalian cell plasma membranes. Combining atomistic-scale molecular dynamics (MD) simulations with Langmuir monolayer experiments, we unraveled the mutual miscibility of POPC and POPS lipids and the intricate intermolecular interactions inherent to these membranes as well as the disparities in position and orientation of PC and PS headgroups. Our experiments revealed micrometer-scale miscibility at all mole fractions of POPC and POPS, marked by modest deviations from ideal mixing with no apparent microscale phase separation. The MD simulations, meanwhile, demonstrated that these deviations were due to strong electrostatic interactions between like-lipid pairs (POPC–POPC and POPS–POPS), culminating in lateral segregation and nanoscale clustering. Notably, PS headgroups profoundly affect the ordering of the lipid acyl chains, leading to lipid elongation and subtle PS protrusion above the zwitterionic membrane. In addition, PC headgroups are more tilted with respect to the membrane normal, while PS headgroups align at a smaller angle, making them more exposed to the surface of the mixed PC/PS membranes. These findings provide a detailed molecular-level account of the organization of mixed PC/PS membranes, corroborated by experimental data. The insights gained here extend our comprehension of the physiological role of PSs.

## Introduction

Cell membranes are an integral part of the architecture of every living cell, playing a pivotal role in both establishing the unique identity of the cell and defining a multitude of internal compartments. These membranes are characterized by their rich lipid composition, often encompassing hundreds of distinct lipid species distributed asymmetrically between the bilayer leaflets.^1−3^ The primary constituents of eukaryotic cell membranes are glycerophospholipids, mainly phosphatidylcholines (PCs), phosphatidylethanolamines (PEs), phosphatidylserines (PSs), and phosphatidylinositols. They make up the majority of lipids found in these membranes, accounting for around 70% of all lipids in mammalian cells.^4,5^ Therefore, glycerophospholipids are largely responsible for the properties of the lipid bilayer that comprise the cell membrane. In addition, they are densely populated with proteins, which collectively cover about 30% of the total membrane surface area. The remarkable heterogeneity of cellular membranes, marked by a nonuniform lateral distribution of lipids and proteins, leads to the formation of distinct nanodomains. These nanodomains play a pivotal role in numerous cellular processes, including membrane fusion, protein trafficking, and signal transduction, among others.^6−9^ In this context, the interplay between lipids and proteins emerges as a captivating and dynamic mechanism, wherein lipids are transported by proteins and proteins, in turn, are modulated by lipids,^10−12^ shedding light on the intricacies of cellular regulation and function.

An important feature of the cell membranes is the asymmetric distribution of lipids between the inner and outer leaflets.^13^ The extracellular leaflet of the plasma membrane of mammalian cells contains predominantly PCs, sphingomyelins, and gangliosides, while the intracellular leaflet is enriched with PEs, PSs, and other anionic lipids. Notably, PSs, which are the main negatively charged lipids in animals, account for 8–15 mol % of all phospholipids in cells.^14^ The asymmetric distribution of PSs is maintained by the type-IV subfamily of P-type ATPases that are responsible for removing PSs from the outer leaflet of the cell membrane.^15^ However, this balance can be disrupted when, on occasion, membrane receptors, known as scramblases, are activated and facilitate the transfer of lipids between membrane leaflets.^16^ The appearance of PSs on the surface of mammalian cells is extremely important for several physiological processes. For example, PS exposed on the plasma membrane regulates the recognition and engulfment of apoptotic cells and modulates key biochemical reactions related to blood clotting, bone mineralization, and various cell–cell interactions.^17^ Abnormal distribution and levels of PSs can lead to blood-related diseases such as sickle cell anemia and congenital bleeding disorder.^18^ In addition, PSs directly interact with and regulate the functions of several proteins, such as protein kinase C, Raf-1 (a serine-threonine kinase), AMPA (a glutamate receptor), and proteins related to exocytosis.^19^

To comprehensively understand the physiological processes triggered by PS, it is necessary to understand the behavior of this negative lipid in the outer leaflet of the mammalian plasma cell membrane. Mixed PC/PS lipid bilayers can serve as models of an extracellular leaflet with exposed phosphatidylserine.^20^ Several studies of PC/PS mixtures have been carried out by using experimental methods and molecular dynamics (MD) simulations. Most of them addressed lipid flip-flop transitions^7,21^ and interactions of anionic/zwitterionic lipid bilayers with mono- and divalent cations^22−24^ or with proteins.^5,25^ In contrast, studies of the molecular organization of PS in mixed membranes are rather scarce. Fragneto et al. studied dipalmitoylphosphatidylcholine (DPPC) with 10 mol % of the negatively charged dipalmitoylphosphatidylserine (DPPS) using neutron reflectivity measurements.^26^ They determined the thickness of the headgroup region to be 0.9 ± 0.1 nm and that of the hydrocarbon region to be 3.6 ± 0.1 nm. Ge et al. used an electron spin resonance (ESR) method to study mixtures of 1-palmitoyl-2-oleoyl-phosphatidylcholine (POPC) and phosphatidylserine (POPS) in the liquid crystalline phase to characterize the effect of headgroup mixing on the structural and dynamic properties of the acyl chains.^27^ The results showed that mixing these lipids results in a significant reduction in the order parameter at the terminal part of the acyl chains and the rotational diffusion coefficient, especially for an equimolar mixture of these lipids. In addition to the experimental studies, MD simulations were used to study the PC/PS bilayers. Jurkiewicz et al. used Berger united-atom MD simulations to investigate the hydration and structures of 4:1 PC/PS bilayers in the environment of monovalent cations (a concentration of 1 M).^20^ The simulations showed deep penetration of the cations into the glycerol level of the lipid bilayer, where they pair with oxygen atoms of the lipid carbonyl groups. The cations combine neighboring lipids to form clusters of up to 4 lipid molecules, which reduces the area per lipid, thickens the membrane, causes lipid headgroups to float, and hinders lipid dynamics. The order parameters of the PS lipid headgroup and glycerol backbone C–H bond order parameters for pure PS and mixed PS/PC lipid bilayers were discussed by Antila et al.^28^ Based on the experimentally measured order parameters (using nuclear magnetic resonance (NMR) spectroscopy), the authors evaluated the quality of the headgroup structures and ion binding affinity in the available MD simulation models of PS lipids.

The above brief review of the literature on mixed PC/PS membranes indicates that the molecular-scale picture of how PS lipids are arranged in lipid bilayers is poorly recognized. In this study, we focused on the molecular organization of the mixed PC/PS bilayers, which represents a simplified model of the outer leaflet of the plasma membrane with exposed PS. Phospholipids with the same acyl chains (2-oleoyl-1-palmitoyl-*sn*-glycero-3-phosphocholine, POPC, and 2-oleoyl-1-palmitoyl-*sn*-glycero-3-phospho-l-serine, POPS) were used to separate the effects of different headgroups on the membrane organization. Our primary objectives are twofold: (i) to elucidate the mutual miscibility of these lipids at the nanoscale and the intricate intermolecular interactions inherent to these membranes and (ii) to assess disparities in the position and orientation of PC and PS headgroups. To this end, we used atomic-scale MD simulations augmented by Langmuir monolayer experiments and Brewster angle microscopy (BAM) to study POPC/POPS bilayers with various compositions. The Langmuir monolayer and BAM measurements allowed us to determine the thermodynamic parameters governing the mixing of PS and PC and revealed the morphological details of the mixed lipid films. Synergistically, the MD simulations were performed to gain a molecular-level understanding of the interactions between the PC and PS headgroups that cannot be directly accessed via experimental approaches. Our results are expected to be extremely useful in understanding the biological functions of phosphatidylserines, as knowledge of the special arrangement of PSs in cell membranes will bring us one step closer to the still elusive understanding of the sensing of surface-exposed phosphatidylserines.

## Materials and Methods

### Materials

**Figure 1:**
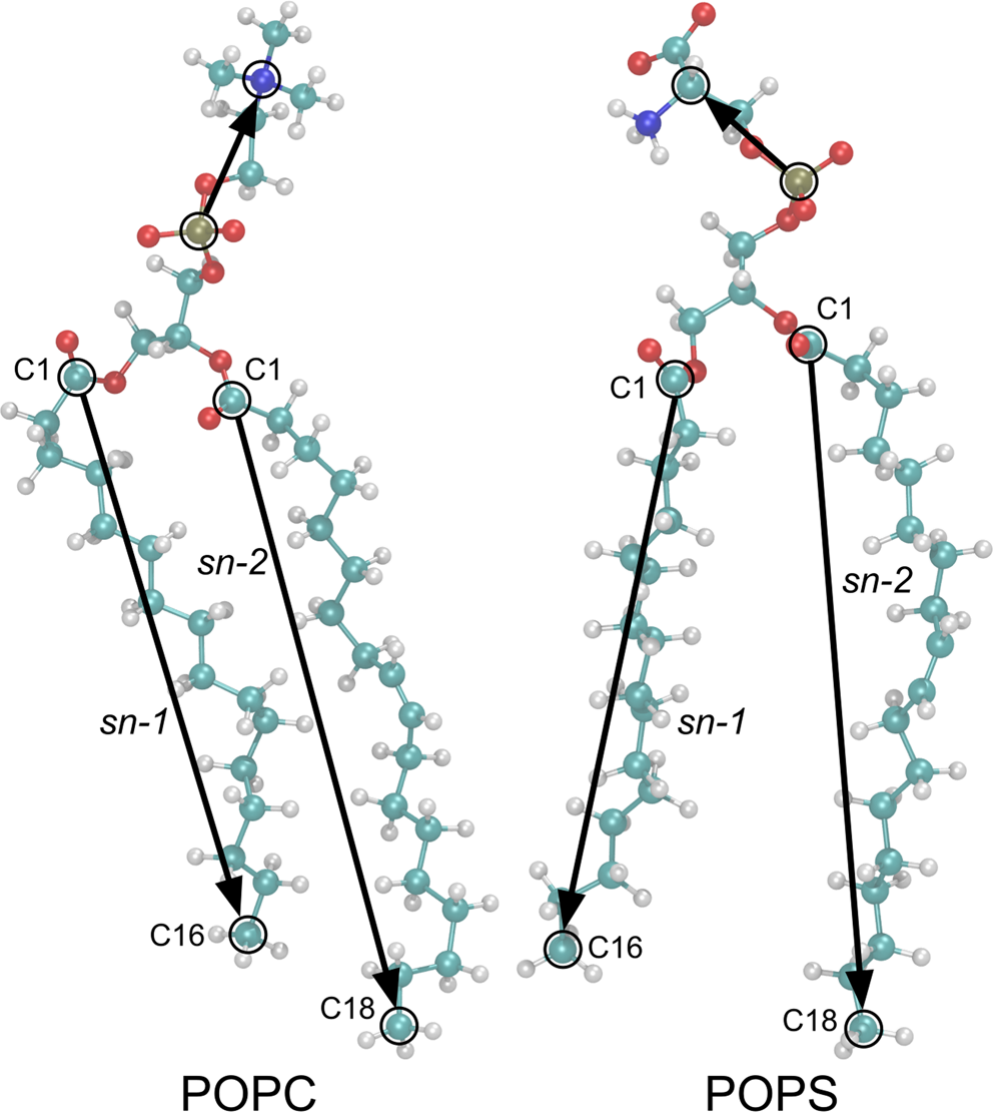
Chemical structures of POPC and POPS (atom color coding is as follows: C: cyan, O: red, N: blue, P: gold, and H: white). Headgroup and lipid chain vectors used in the orientation analysis are shown as black arrows.

2-Oleoyl-1-palmitoyl-*sn*-glycero-3-phosphocholine (POPC), 2-oleoyl-1-palmitoyl-*sn*-glycero-3-phospho-l-serine (sodium salt, POPS) (Figure 1), chloroform, methanol, and phosphate-buffered saline (PBS, tablets) were obtained from Sigma-Aldrich. Ultrapure Milli-Q water was used in the experiments.

### Monolayer Experiments

The experiments were carried out with a KSV 2000 Langmuir trough (KSV Instruments Ltd., Helsinki, Finland) equipped with an ultraBAM (Accurion GmbH, Goettingen, Germany) microscope, as previously described.^29^ The surface pressure was measured with an accuracy of ±0.1 mN/m using a Wilhelmy plate made of filter paper (ashless Whatman Chr1) connected to an electrobalance. Lipid stock solutions were prepared in a mixture of chloroform/ethanol (9:1 v/v) and kept refrigerated at −20 °C. The lipid mixtures were deposited onto the 1 mM phosphate-buffered saline (PBS) solution subphase with a Hamilton analytical syringe. After spreading, the monolayers were left for 5 min and then compressed with a barrier speed of 10 mm/min. The experiments were performed at 23 °C (the subphase temperature was controlled using a Julabo water circulating bath). All of the experiments were repeated at least twice to ensure consistent results.

## MD Simulations

### Simulated Systems

All-atom MD simulations were conducted for lipid bilayers composed of POPC and POPS mixtures with varying *X*_POPS_ values (0.0, 0.2, 0.4, 0.6, 0.8, and 1.0). The chemical structures of the lipids can be found in Figure 1, and a comprehensive description of the components of all simulated systems is provided in Table 1. The simulations were carried out at 22 °C.

**Table 1:** Detailed Description of the Compositions of the Simulated Systems

system	POPC	POPS	K^+^	Cl^–^	water	# of repeats × simulation length
POPC	200	0	22	22	10,000	3 × 1 μs
POPC/POPS, *X*_POPS_ = 0.2	160	40	62	22	10,000	3 × 1 μs
POPC/POPS, *X*_POPS_ = 0.4	120	80	102	22	10,000	3 × 1 μs
POPC/POPS, *X*_POPS_ = 0.6	80	120	142	22	10,000	3 × 1 μs
POPC/POPS, *X*_POPS_ = 0.8	40	160	182	22	10,000	3 × 1 μs
POPS	0	200	222	22	10,000	3 × 1 μs

The CHARMM-GUI tool was used to construct the initial structures of the lipid membranes.^30^ After the addition of 0.15 M KCl and solvation, the systems were subjected to energy minimization using the steepest descent algorithm. Subsequently, 1-μs MD simulations in the isobaric–isothermal (NpT) ensemble were performed. The first 900 ns of the simulations was considered as an equilibration period, and thus, only the last 100 ns of each trajectory was analyzed unless otherwise specified. Each simulation was repeated three times with different initial configurations of lipids, ions, and water molecules.

### Simulation Parameters

The parametrization of lipid molecules was performed using the CHARMM36m force field.^31^ The TIP3P model^32^ was employed to describe the behavior of water molecules. During the simulations, a constant pressure of 1 bar was maintained using the Parrinello–Rahman algorithm,^33^ with a pressure constant of 5 ps. The pressure was controlled independently along the bilayer normal (*z*-axis) and in the bilayer plane (*xy*-plane) using a semi-isotropic algorithm. The temperature of the lipids and solvent (water and ions) was regulated separately using the Nosé–Hoover algorithm,^34^ with a temperature constant of 1 ps. Periodic boundary conditions were applied in all three dimensions. Covalent bonds were preserved using the LINCS algorithm,^35^ with a simulation time step of 2 fs. The SETTLE method^36^ was employed for water molecules. Long-range electrostatic interactions beyond a cutoff of 1.2 nm were treated using the particle mesh Ewald algorithm.^37,38^ The GROMACS 2022.3 software package^39^ was used for all MD simulations, and Visual Molecular Dynamics (VMD) software^40^ was used to visualize the simulated systems.

### Analysis of MD Simulations

#### Interaction Energies (Miscibility Energetics)

The interaction energies were calculated by using the “gmx energy” tool of the GROMACS 2022.3 software package. Electrostatic (Coulomb) and dispersive (van der Waals) interactions were calculated separately for each lipid–lipid interaction (POPC–POPC, POPS–POPS, and POPC–POPS). The energy values were averaged over the analyzed trajectory length, the number of repeats, and the total number of lipid molecules in the system.

#### Tilt Angles

For each lipid type, tilt angles were measured between the *z*-axis of the system (the normal to the bilayer surface) and a vector defined within one of the three lipid molecule segments: polar headgroups and two acyl chains (*sn*-1 and *sn*-2). The headgroup vector was spanned by P and N atoms for POPC and P and C atoms for POPS. The chain vectors were the same for both lipid types: C1–C18 for *sn*-1 and C1–C16 for *sn*-2. The defined vectors are schematically presented in Figure 1. All tilt angles were calculated by using the “gmx bundle” tool (GROMACS 2022.3). It is worth noting that for POPS, the P–C vector employed is not the only possible choice, and there are alternatives, such as the P–N vector, combining the P atom and the N atom of the amino group. Further analysis of the vector choice can be found in the SI (Figure S1).

#### Order Parameter (*S*_CH_)

The order parameters were calculated for the *sn*-1 acyl chain for the last 200 ns of the trajectory using the “gmx order” tool (GROMACS 2022.3). The exact definition of *S*_CH_ can be found in ref (41).

#### Membrane Thickness (*d*_P_)

The thickness of the lipid bilayer was estimated using the “gmx distance” tool (GROMACS 2022.3) as the average distance between the phosphorus atoms in the opposite leaflets. Averaging was performed over the analyzed trajectory and the number of repeats.

#### Mass Density Profiles

Mass density profiles were calculated for lipid headgroups, solvents, and ions to illustrate the distribution of system components along the normal to the bilayer. The calculations were performed using the “gmx density” tool (GROMACS 2022.3), and the resulting profiles were plotted along the *z*-axis, where the averaged center of the bilayer was assigned *z* = 0.

#### Contact Analysis

The number of lipid–lipid and lipid–solvent contacts was estimated by using the “gmx mindist” tool (GROMACS 2022.3), with a contact cutoff distance of 0.6 nm. The contacts for each pair of molecules were averaged over the analyzed trajectory length and number of repeats.

#### Area Per Lipid (APL)

To calculate the average in-plane area of POPC and POPS lipids in systems with different *X*_POPS_ values, the APL@Voro lipid bilayer analysis tool was used.^42^ At each step of the analyzed trajectory, Voronoi tessellation was created at the height of the average position of the headgroup phosphorus atoms in the upper and lower leaflets. The area of the resulting polygons was then averaged over the trajectory length and number of repeats for each lipid type and system composition.

## Results and Discussion

We studied mixed POPC/POPS membranes with different POPS mole fractions (*X*_POPS_). Both lipids have the same acyl chains attached to glycerol (Figure 1), which allowed us to separate the effects of different headgroups on the membrane organization. The POPS headgroup consists of a negatively charged phosphate group esterified with serine, having a dissociated carboxyl group (negative charge) and an ammonium group (positive charge) under physiological conditions. The POPC headgroup, on the other hand, is relatively large and zwitterionic (containing a negatively charged phosphate group esterified with choline and a positively charged quaternary ammonium group).

### Monolayer Experiments Indicate Nonideal Mixing between POPC and POPS, but No Phase Separation was Observed at the Microscale

**Figure 2:**
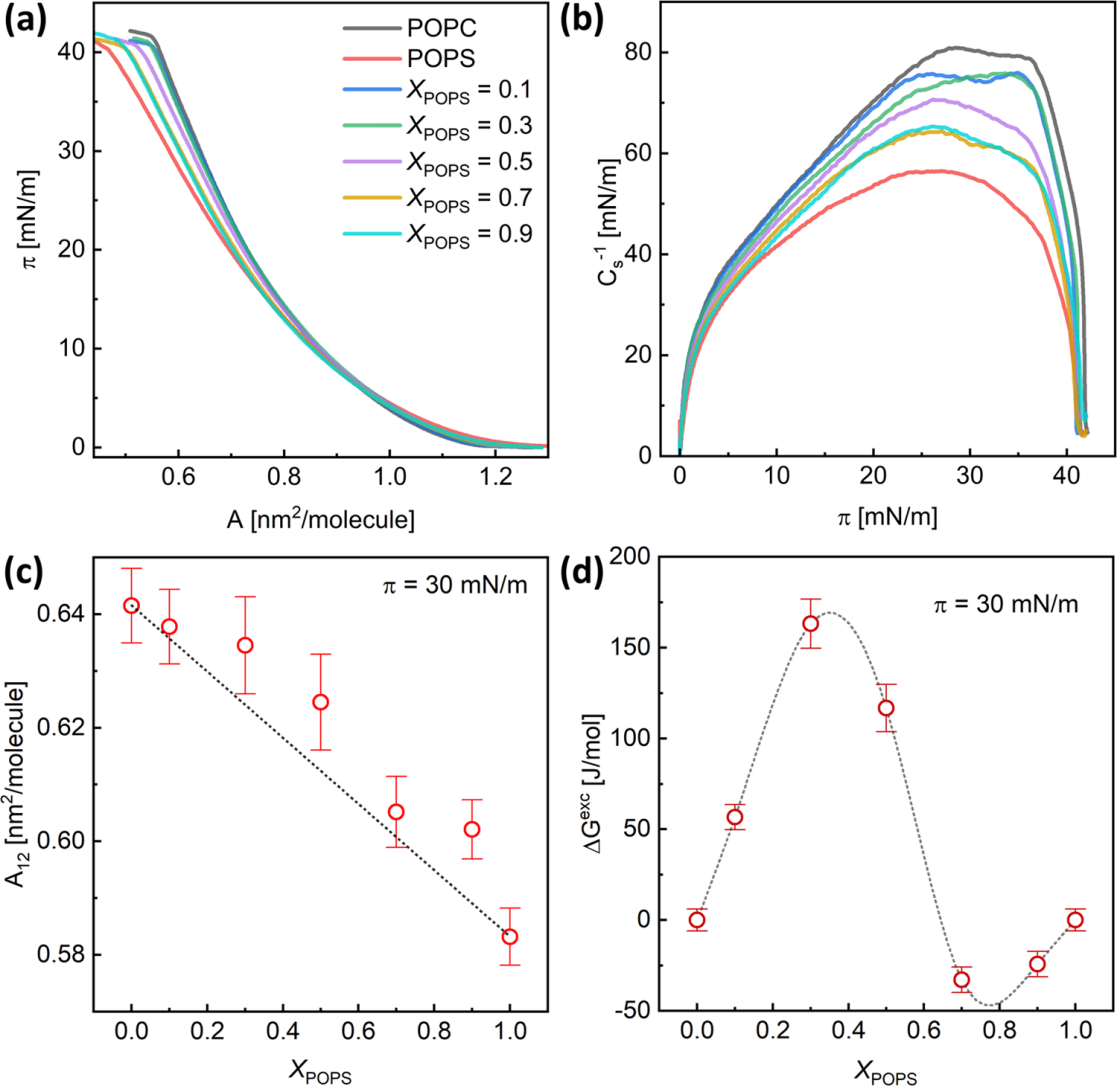
Surface pressure (π)–area (*A*) isotherms (A) and the compression modulus (*C*_s_^–1^) versus π (B) for pure POPC, pure POPS, and mixed monolayers measured at 23 °C. (C) Dependence of the mean area per lipid molecule (*A*_12_) on the composition for the POPC/POPS mixed monolayers at π = 30 mN/m. The dashed straight line corresponds to the ideal mixing of the film components. (D) Values of excess free energy of mixing (Δ*G*^exc^) as a function of the composition of the POPC/POPS monolayers at π = 30 mN/m.

The Langmuir monolayer experiments were used to gain some insight into the interactions between lipids in the POPC/POPS membranes. The properties of monomolecular layers forming at the air/water interface can be determined from the isotherms of surface pressure (π) against area (*A*), which were measured during compression of the lipid film. The shape and position of these isotherms reflect the organization of molecules at the interface and the state of the monomolecular layer.^43^ The π–*A* isotherms, recorded for mixed POPC/POPS films, which were formed on a subphase of 1 mM PBS at 23 °C, are shown in Figure 2a. All of the isotherms exhibit a small slope, characteristic of highly compressible monolayers.

Based on the π–*A* isotherms, the compression modulus (*C*_s_^–1^) was calculated according to the equation
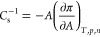
1wherein *A* is the area per molecule at a surface pressure of π. Figure 2b shows the calculated values of the compression modulus (*C*_s_^–1^) as a function of the surface pressure. The maximum value of *C*_s_^–1^ ((*C*_s_^–1^)_max_) increases from 56 mN/m for pure POPS to 81 mN/m for pure POPC. Following the Davies and Rideal classification, monolayers with maximum *C*_s_^–1^ values between 50–100 mN/m are categorized to be in the liquid state.^44^ Thus, all studied monolayers were in the liquid state under our experimental conditions, which aligned with the melting temperatures of both lipid bilayers. The gel-to-liquid crystalline phase transition temperatures for the POPC and POPS bilayers are −2 and 14 °C, respectively.^45^ The decrease in the (*C*_s_^–1^)_max_ value for POPS in comparison to that for POPC shows that the POPS monolayer is slightly more fluid compared to the zwitterionic film.

To assess the miscibility of the monolayer components, the average molecular area (*A*_12_, directly determined from the π–*A* isotherms at a given surface pressure) was plotted as a function of the monolayer composition (Figure 2c). In addition, the excess free energy of mixing (Δ*G*^exc^) was calculated to quantify the strength of the interaction between the molecules in the mixed systems
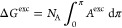
2
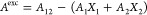
3where *A*_1_ and *A*_2_ are the mean molecular areas of the respective lipids in their pure films at a given surface pressure, *X*_1_ and *X*_2_ are the mole fractions of components 1 and 2 in the mixed film, and *N*_A_ is Avogadro’s number. The excess area *A*^exc^ is the difference in the measured area per molecule of the mixture (*A*_12_) and the weighted average molecular areas of the constituent molecules (*A*_1_ and *A*_2_).

The analysis was performed at π = 30 mN/m since, at these conditions, the properties of lipid monolayers correlate with those of bilayers.^46^ The area per lipid values for POPC and POPS at 23 °C, as determined from the isotherms for pure lipids, are 0.64 ± 0.01 and 0.58 ± 0.01 nm^2^, respectively. The values for POPC are consistent with those obtained from other experimental methods. Konig et al. reported the area per lipid of 0.64 ± 0.01 nm^2^ for fully hydrated POPC at 25 °C.^47^ The APL for POPC was determined using solid-state ^13^C NMR spectroscopy to be 0.604 ± 0.036 nm^2^ at 28 °C and 0.705 ± 0.042 nm^2^ at 48 °C.^48^ In the case of POPS, there are somewhat greater discrepancies in the reported APL values. Using MD simulations (CHARMM C36 lipid parameters), Venable et al. calculated the APL value of POPS in 0.1 M NaCl to fall within the range of 0.554–0.584 nm^2^. The structure of the POPS bilayer was previously studied using small-angle neutron (SANS) and X-ray (SAXS) scattering.^49^ The authors reported a lipid area of 0.627 nm^2^ at 25 °C. The discrepancies in the APL values for this lipid likely stem from varying measurement conditions, in particular the different cation content, which strongly influences the behavior of phosphatidylserines.^50^

**Figure 3:**
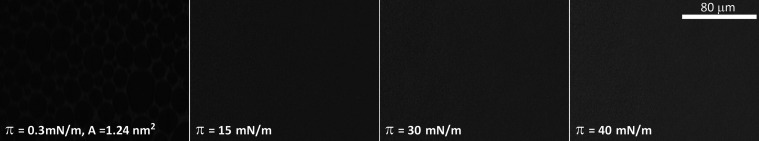
BAM images taken for the POPC/POPS with the *X*_POPS_ = 0.4 monolayer at different stages of compression at 23 °C.

The mean molecular areas determined from the isotherms (*A*_12_) (Figure 2c) surpass those predicted for ideal mixing. They change irregularly with the film composition, but in all cases, expansion of the monolayers can be observed. Such deviations in *A*_12_ values indicate the presence of repulsive forces between the two components in the POPC/POPS monolayers, which may lead to partial miscibility or even phase separation. Nonideal behavior of mixed PC/PS monolayers (primarily expansion) was previously reported for 1-stearoyl-oleoyl-*sn*-glycero-3-phosphocholine and 1-stearoyl-2-oleoyl-*sn*-glycero-3-phospho-l-serine, which was attributed to the existence of PS molecules with bound and unbound counterions.^39^ The values of the excess free energy of mixing (Δ*G*^exc^, Figure 2d) also display irregularity. They are positive for monolayers with *X*_POPS_ ≤ 0.6, indicating interactions of less attractive (compared to monolayers composed of a single lipid type) or repulsive nature. Films with *X*_POPS_ ≥ 0.7 exhibit slightly negative deviations from ideal behavior, suggesting that interactions are more attractive than in monolayers with only one component. The Δ*G*^exc^ values are only marginally positive and do not exceed 0.175 kJ/mol. Such a slight deviation from ideal mixing is unlikely to cause phase separation. We recently suggested that phase separation becomes apparent when Δ*G*^exc^ surpasses the limit of 0.8–1.2 kJ/mol. For positive Δ*G*^exc^ values that remain below this threshold, small lipid aggregates can form.^51^ Indeed, the BAM images (Figure 3) captured for this system show the complete homogeneity of the monolayers, irrespective of film composition. This implies that the POPC and POPS are miscible, at least on the microscale.

### MD Simulations Reveal the Clustering of POPC and POPS Lipids at the Nanoscale

**Figure 4:**
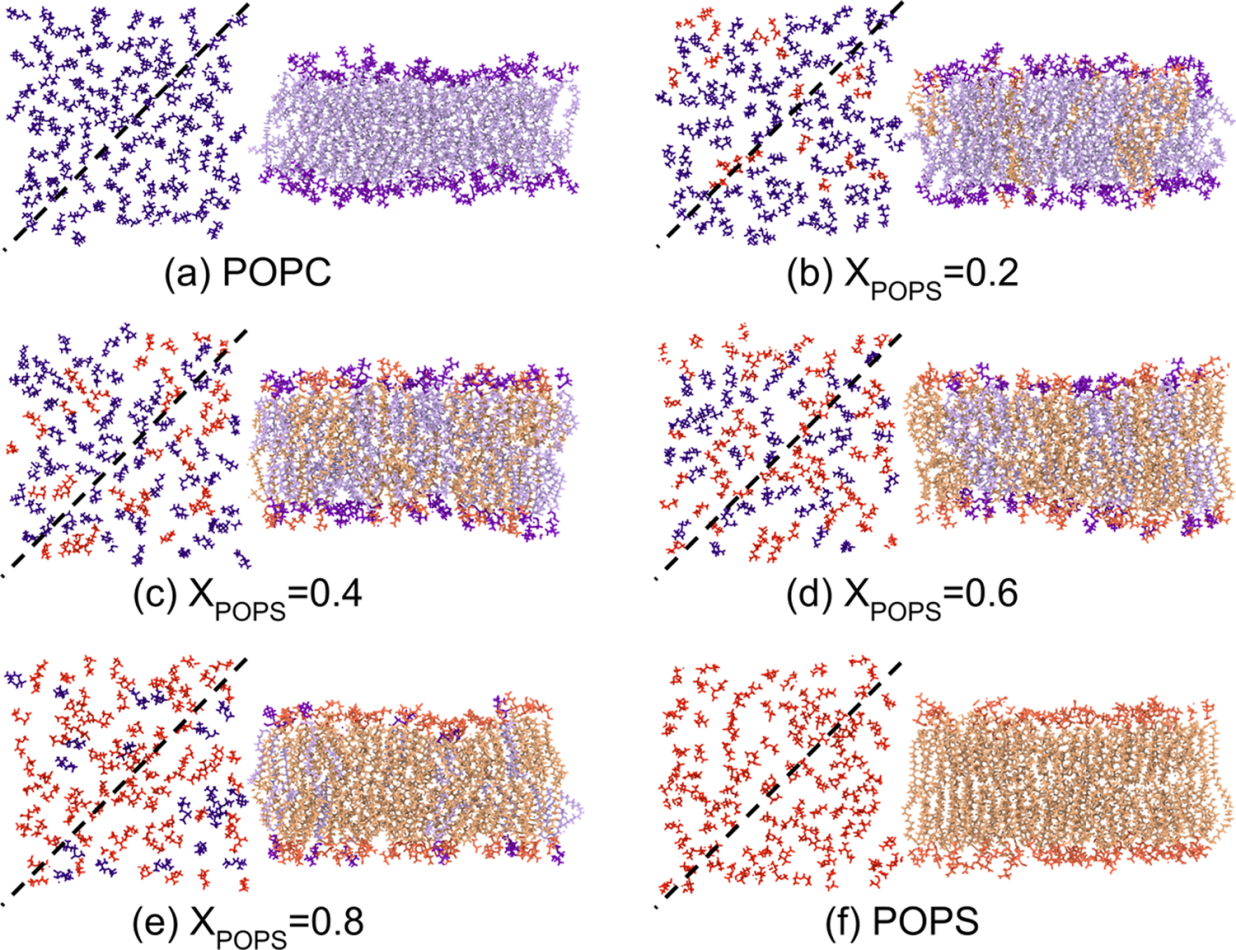
Representative snapshots (top and side views) of the POPC/POPS lipid bilayers with different POPS contents: (a) *X*_POPS_ = 0.0, (b) *X*_POPS_ = 0.2, (c) *X*_POPS_ = 0.4, (d) *X*_POPS_ = 0.6, (e) *X*_POPS_ = 0.8, and (f) *X*_POPS_ = 1.0. POPC is shown in light purple with the headgroups in dark purple, and POPS is shown in orange with the headgroups in red. For clarity, the top view depicts the lipid headgroups from the upper leaflet only. The side view depicts the diagonal cross-section of the lipid bilayer, as indicated by the black dashed line. Water molecules and ions are not shown for clarity.

We performed MD simulations of mixed POPC/POPS bilayers with varying POPS mole fractions (*X*_POPS_). Figure 4 shows the representative snapshots (both top and side views) captured at the end of simulations for bilayers with different POPS contents. These snapshots indicate a slight inclination of the two lipids to form small clusters of like lipids, especially evident in systems with *X*_POPS_ = 0.4 and 0.6. However, there is no observation of phase separation in either system. To further analyze the mutual miscibility of these lipids, we calculated the energy of interactions in like pairs (POPC–POPC and POPS–POPS) and mixed pairs (POPC–POPS), as well as the number of contacts between the lipid molecules.

### Due to the Substantial POPC–POPC and POPS–POPS Electrostatic Attractions, Interactions in Like Pairs Are More Robust Compared to Mixed Pairs

**Figure 5:**
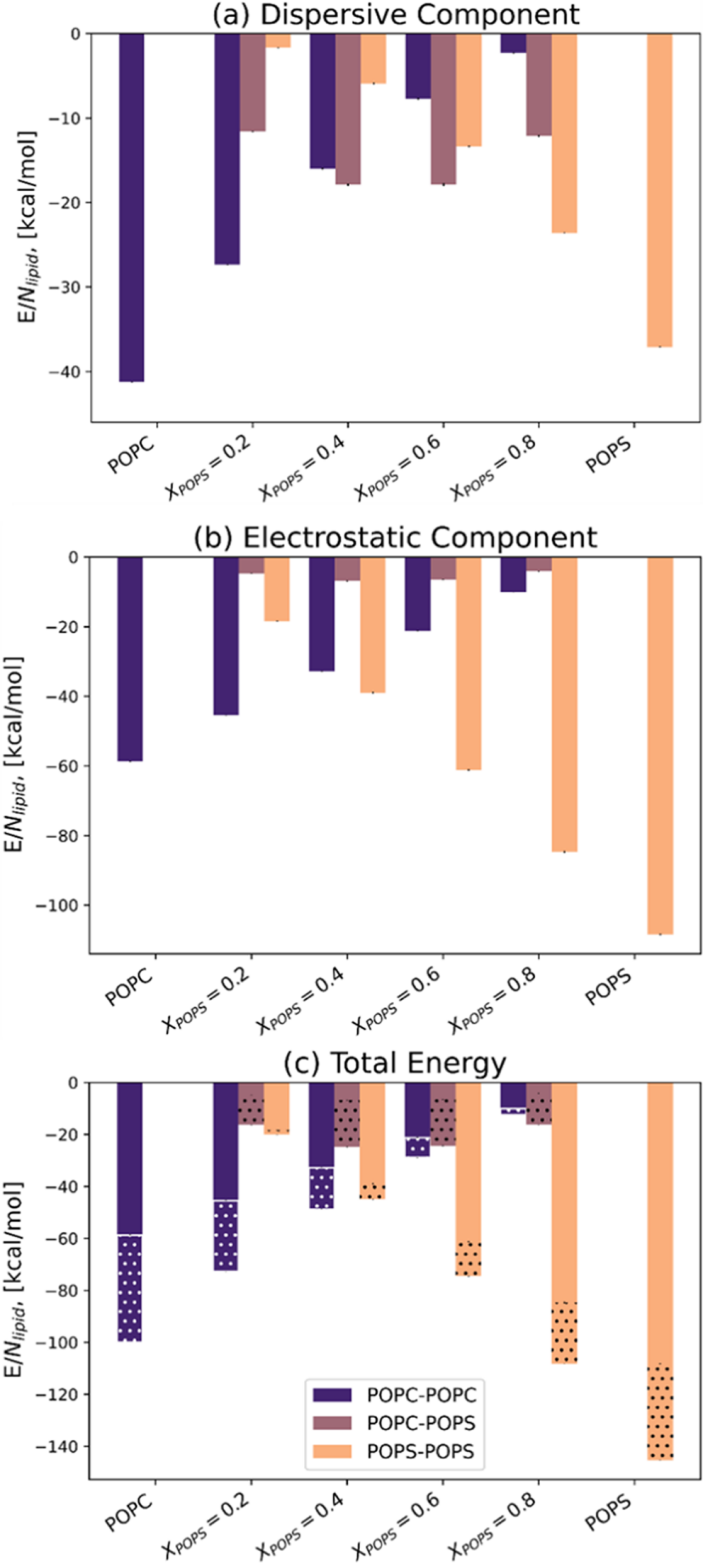
Dispersive (a) and electrostatic (b) components of interaction energy and total interaction energy (c) between lipid molecules in the POPC/POPS bilayers as a function of the POPS mole fraction (*X*_POPS_). The dotted area shows the dispersive component. All energies were calculated as averages over time and the number of repeats and normalized by the total number of lipids.

The miscibility between the lipid membrane components depends on the interactions between them. Therefore, we calculated the interaction energies for like pairs and mixed pairs in the POPC/POPS bilayers, separating the electrostatic and dispersive components (Figure 5). The dispersive component includes all of the terms of the Lennard–Jones interaction potential, that is, the dispersive energy (acting at large interatomic distances and always attractive) and the Pauli repulsion (acting at much smaller interatomic distances). The electrostatic component describes the Coulomb interactions between the partial charges localized at the atomic positions. The energy values were normalized by the number of lipids in the system. For pure membranes, the dispersive component is similar for both lipids (Figure 5a). However, the total interaction energy between POPS molecules falls below that of POPC (Figure 5c) due to the stronger electrostatic interactions between the serine headgroups (Figure 5b). In the case of mixed systems, the electrostatic component is dominant for like pairs, while the dispersive energy is lower for POPC–POPS interactions. In addition, for *X*_POPS_ = 0.4, the absolute value of the total energy of POPC–POPS interactions is nearly half that of like pairs, indicating energetically unfavorable mixing of these two lipids. This is consistent with our measurements of POPC/POPS monolayers (the maximum in the Δ*G*^exc^ profile for *X*_POPS_ = 0.4, Figure 2d). In contrast, the overall interactions in mixed pairs within the membrane with *X*_POPS_ = 0.8 surpass those in POPC–POPC interactions, primarily due to the dispersive component. Consequently, the mutual miscibility of these two lipids in this composition is anticipated to be favorable, corresponding well to the minimum in the dependence of the excess free energy of mixing on the POPC/POPS monolayer composition (Figure 2d).

### Number of Contacts Indicates That in Mixed Membranes, Both POPS and POPC Tend to Cluster with Lipids of the Same Type

**Figure 6:**
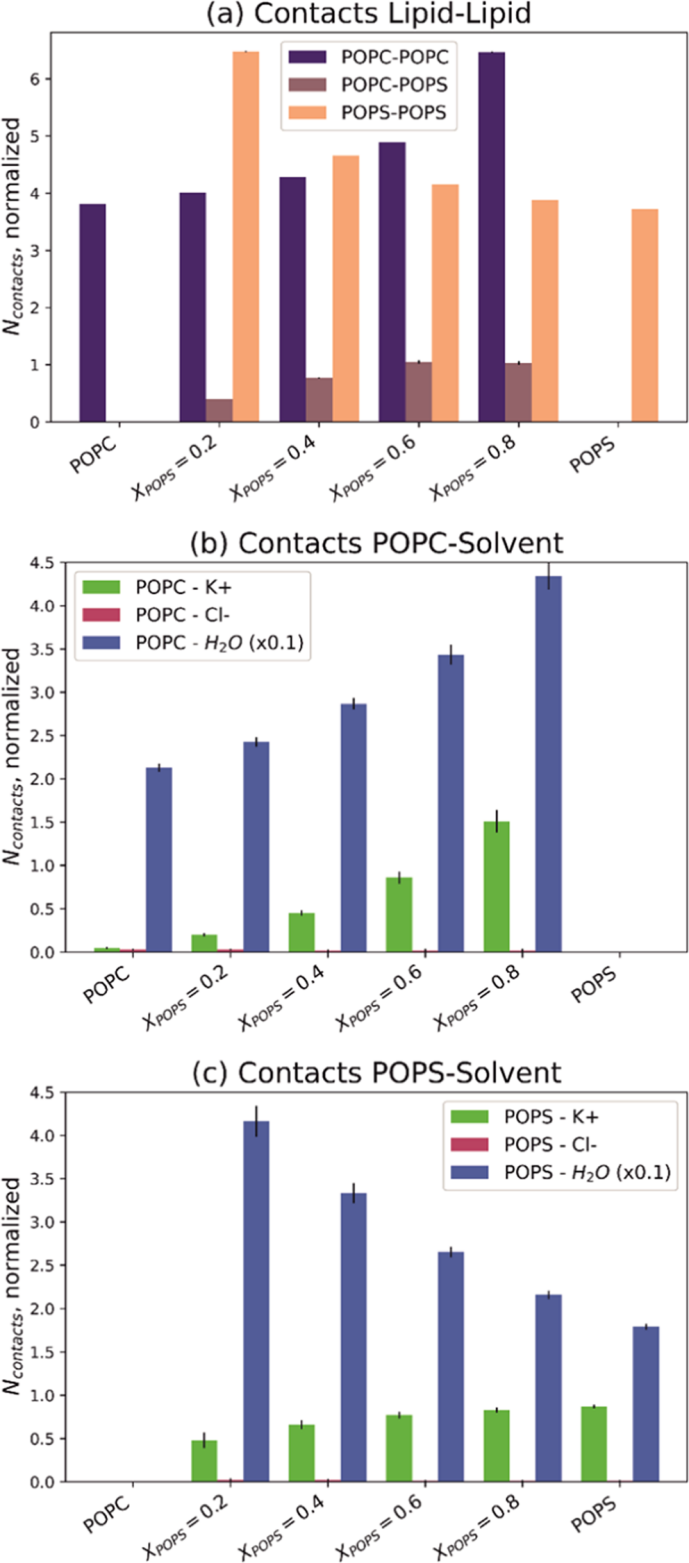
Average number of lipid–lipid (a) and lipid–solvent contacts ((b) for POPC, (c) for POPS). The number of contacts for each pair of molecules was averaged over the analyzed trajectory length and the number of repeats.

To gain further insight into the lipid interactions, we calculated the average number of contacts (Figure 6) and normalized it by the number of lipids in the system. The average number of lipid–lipid interactions in pure membranes is similar for both POPC and POPS, with approximately 4 contacts per lipid (Figure 6a). In mixed bilayers, the number of POPC–POPC and POPS–POPS contacts increases as the concentration of a given lipid decreases, reaching a maximum value of about 6. This signals a trend for both POPC and POPS to cluster with like lipids. This is further corroborated by the low number (≤1) of POPC–POPS contacts.

The lipid–solvent interactions (Figure 6b,c) suggest that the pure POPC membrane is slightly more hydrated compared with the pure POPS bilayer. This trend persists in the case of mixed bilayers, where POPC has slightly more contact with water. This is consistent with the order parameter analysis described below, which suggests that the POPS bilayer is more ordered and, thus, less permeable to water molecules. The average number of POPC–K^+^ interactions increases with an increasing POPS concentration in the system (Figure 6b). This can be attributed to the growing surface charge density of the membrane (with increasing POPS concentration), which attracts potassium ions to the surface of the membrane, consequently increasing the likelihood of POPC–K^+^ interactions. On average, the POPS molecule interacts with only one potassium ion (Figure 6c).

To further corroborate the contact analysis presented above and remove the cutoff dependency needed in the number of contacts analysis, we calculated the radial distribution functions (RDFs) and cumulative RDFs for POPC–POPS pairs in the mixed lipid bilayers with *X*_POPS_ = 0.2, 0.4, 0.6, and 0.8. These are presented in Figure S2.

### MD Simulations Provide a Nanoscale Perspective on the Structure of the POPC/POPS Bilayer

To elucidate the lipid arrangement in the POPC/POPS bilayers, we calculated APL, *d*_P_, *S*_CH_, and tilt angles of headgroups and hydrocarbon chains for each membrane. The APL values were calculated using the Voronoi tessellation method,^33^ separately for each lipid (Table 2). The results show that the average area per PC is larger than the average area per PS. Meanwhile, the addition of POPS to the POPC bilayer reduces the average area per PC from 0.634 nm^2^ in the pure POPC bilayer to 0.608 nm^2^ in the system with *X*_POPS_ = 0.8. The opposite effect can be observed for the average area per PS, where the addition of POPC to the POPS bilayer increases the average area per PS from 0.563 nm^2^ in the pure POPS bilayer to 0.597 nm^2^ in the system with *X*_POPS_ = 0.2. The thickness of the bilayer was calculated as the average distance between the phosphorus atoms in the opposite leaflets (Table 2). The *d*_P_ value for pure POPC is smaller compared to that of the pure POPS system (which is consistent with APLs). The increase in POPS content in the mixed membrane systematically increases its thickness.

**Table 2:** Values of Area Per Lipid (APL) and Membrane Thickness (*d*_P_)^a^

system	APL for POPC [nm^2^]	APL for POPS [nm^2^]	*d*_P_ [nm]
POPC	0.634		3.92
POPC/POPS, *X*_POPS_ = 0.2	0.622	0.597	4.00
POPC/POPS, *X*_POPS_ = 0.4	0.616	0.584	4.06
POPC/POPS, *X*_POPS_ = 0.6	0.605	0.574	4.13
POPC/POPS, *X*_POPS_ = 0.8	0.608	0.569	4.19
POPS		0.563	4.25

a The errors (standard deviations of the averages) were smaller than 0.007 nm^2^ and 0.05 nm for the APL and *d*_P_ estimation, respectively.

**Figure 7:**
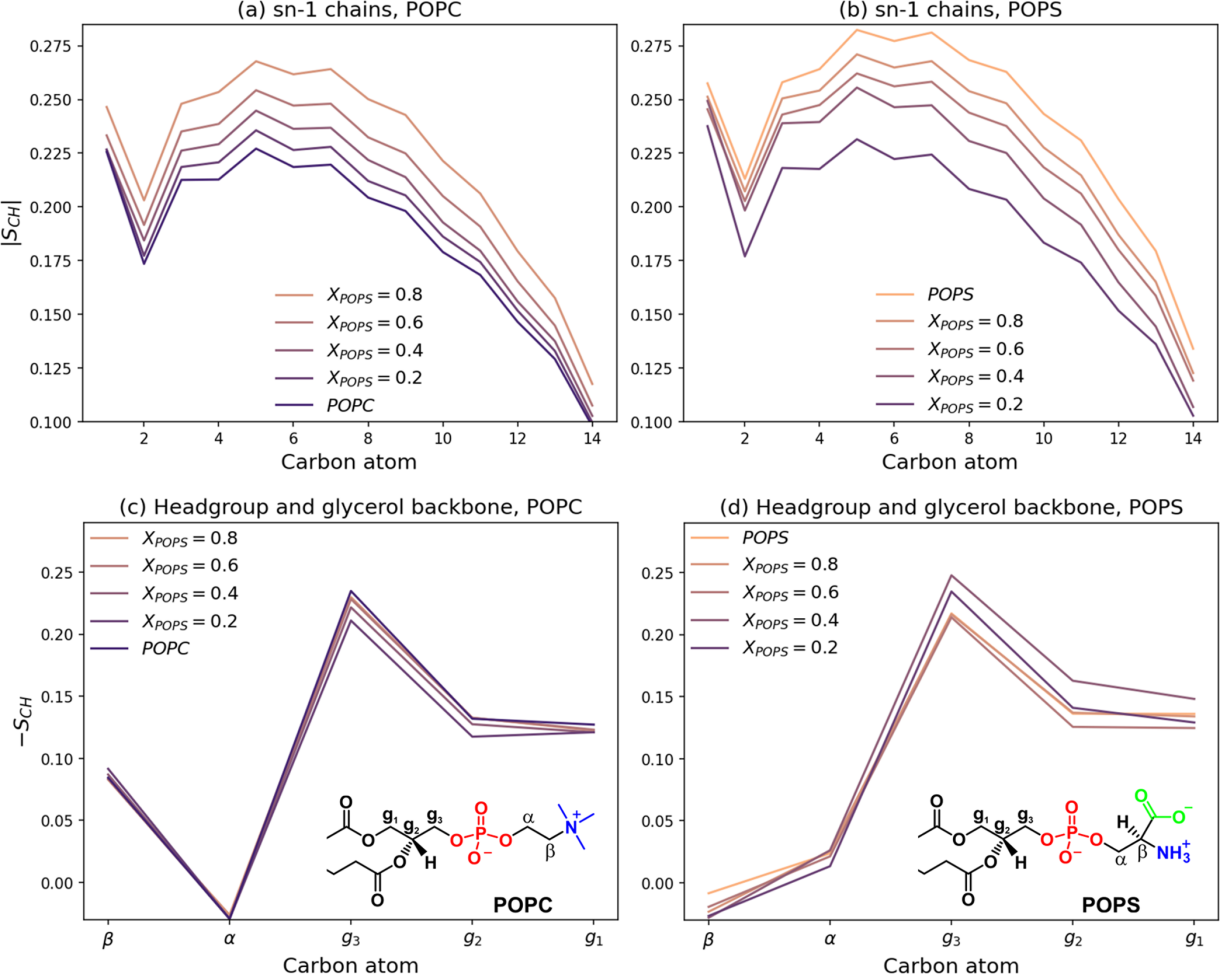
Hydrogen order parameter (*S*_CH_) profiles of the *sn*-1 chains for POPC (a) and POPS (b) in the POPC/POPS bilayers as a function of the POPS mole fraction (*X*_POPS_). Headgroup and glycerol backbone order parameter profiles for POPC (c) and POPS (d) in the POPC/POPS bilayers as a function of *X*_POPS_. The insets show the chemical structures and labels of the carbons of the headgroups and glycerol backbones. For comparison with the experimental data, please refer to Figure 2 in ref (17).

We then investigated the arrangement of lipid acyl chains in the POPC/POPS bilayers. Figure 7a,b illustrates the profiles of the hydrogen order parameter (|*S*_CH_|) along the *sn*-1 chains of both POPC and POPS lipids. Comparing the corresponding |*S*_CH_| values, the pure POPC bilayer displays lower order parameter values compared with the pure POPS system, indicating that the POPC membrane is less ordered. This underscores the substantial influence of the headgroup on the arrangement of attached hydrocarbon chains. With increasing POPS concentration in the POPC/POPS bilayer, the overall ordering of the membrane increases. The heightened ordering results in an elongation of the lipid acyl chains, which is consistent with the decrease in the APL and increase in the *d*_P_ described above. Our results are consistent with experimental findings. The order parameters for the POPC membrane were previously determined by NMR spectroscopy.^37^ The maximum value of |*S*_CH_| was found to be 0.21 at 28 °C for segments of the palmitoyl chain near the headgroup and gradually decreases toward the methyl end of the hydrocarbon chain.

In addition, we calculated the order parameters for the lipid headgroups (choline and serine for POPC and POPS, respectively) and the C–H bonds of the glycerol backbone for both lipid species in the mixed membranes. As depicted in Figure 7c, the ordering within the POPC headgroup (carbons α and β depicted in the insets of Figure 7c) remains almost unchanged with respect to the composition of the mixed membrane, while the glycerol backbone of this lipid becomes slightly stiffened when mixed with the anionic lipid. Notably, the introduction of POPS lipids into the POPC bilayers makes the PS glycerol group (carbons g_1_, g_2_, and g_3_) noticeably more rigid (Figure 7d), while the ordering within serine (carbons α and β) decreased slightly. The order parameters of headgroups and glycerol backbones of POPS and POPC have been previously determined experimentally by ^2^H NMR.^17,52^ While acknowledging that prior molecular dynamics models for PS and PC did not comprehensively produce quantitative agreement with the experimental data,^17^ our simulations notably follow the experimentally observed trends. In particular, the *S*_CH_ values derived from our simulations closely follow the experimental trends for POPC (for comparison, refer to Figure 2 in ref (17), black square data set). In the case of the anionic lipid, the CHARMM36m model shows somewhat reduced performance, although it remains capable of accurately reproducing the order parameters for the α, g_1_, and g_3_ carbon atoms. It is noteworthy that the CHARMM36m force field used in our calculations has previously shown predictive ability in terms of headgroup and glycerol group order parameters, showing the best agreement with experiments compared to other MD models (refer to red circle data set in Figure 2, ref (17).).^17^

### In the Mixed Membranes, POPS Headgroups Are Located Slightly Shallower than POPC Headgroups

**Figure 8:**
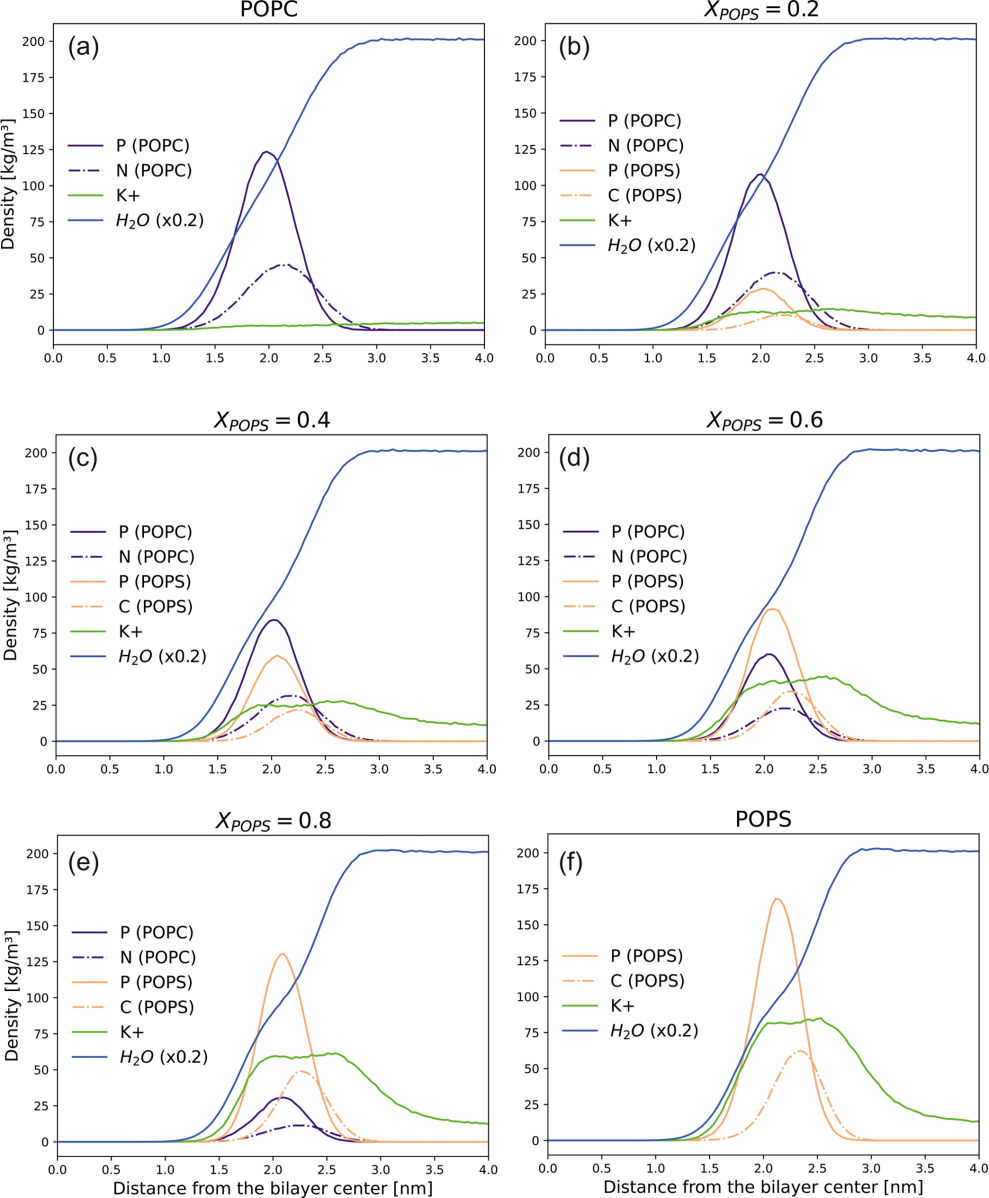
Mass density profiles across the lipid membrane for selected lipid atoms, water (blue line), and K^+^ (green line) in lipid bilayers with varying POPS content: (a) *X*_POPS_ = 0.0, (b) *X*_POPS_ = 0.2, (c) *X*_POPS_ = 0.4, (d) *X*_POPS_ = 0.6, (e) *X*_POPS_ = 0.8, and (f) *X*_POPS_ = 1.0. For POPC, phosphorus (violet solid line) and nitrogen (violet dashed-dotted line) are depicted. For POPS, the phosphorus (orange solid line) and carbon atom (orange dash-dotted line) attached to the ammonium group are shown. For clarity of the presentation, the mass density profile of water was scaled down by 0.2.

To assess the depth at which lipid headgroups are positioned as well as the depth of water and potassium ions penetration into the mixed bilayers, mass density profiles across the membrane of selected lipid atoms (phosphorus and nitrogen atoms for POPC, and phosphorus and carbon atoms attached to the ammonium group for POPS), water, and K^+^ were calculated (Figure 8). In both lipid types, the distributions of phosphate groups are single symmetrical peaks. Notably, the POPS phosphate groups are found to be located somewhat shallower in the bilayer compared to POPC. In addition, the mass density profiles indicate that the depth of water penetration decreases gradually as the POPS content in the mixed membrane increases. Consequently, the POPS membrane is less hydrated than the zwitterionic one. The distributions of K^+^ counterions exhibit two maxima at ca. 2.0 and 2.7 nm. This indicates the preference of potassium cations to be located at the water–membrane interface or potentially penetrate the polar region of the POPC/POPS membrane in proximity to the phosphates.

### PC Headgroups are Tilted about 66° to the Membrane Normal, while PS Headgroups are Less Tilted

**Figure 9:**
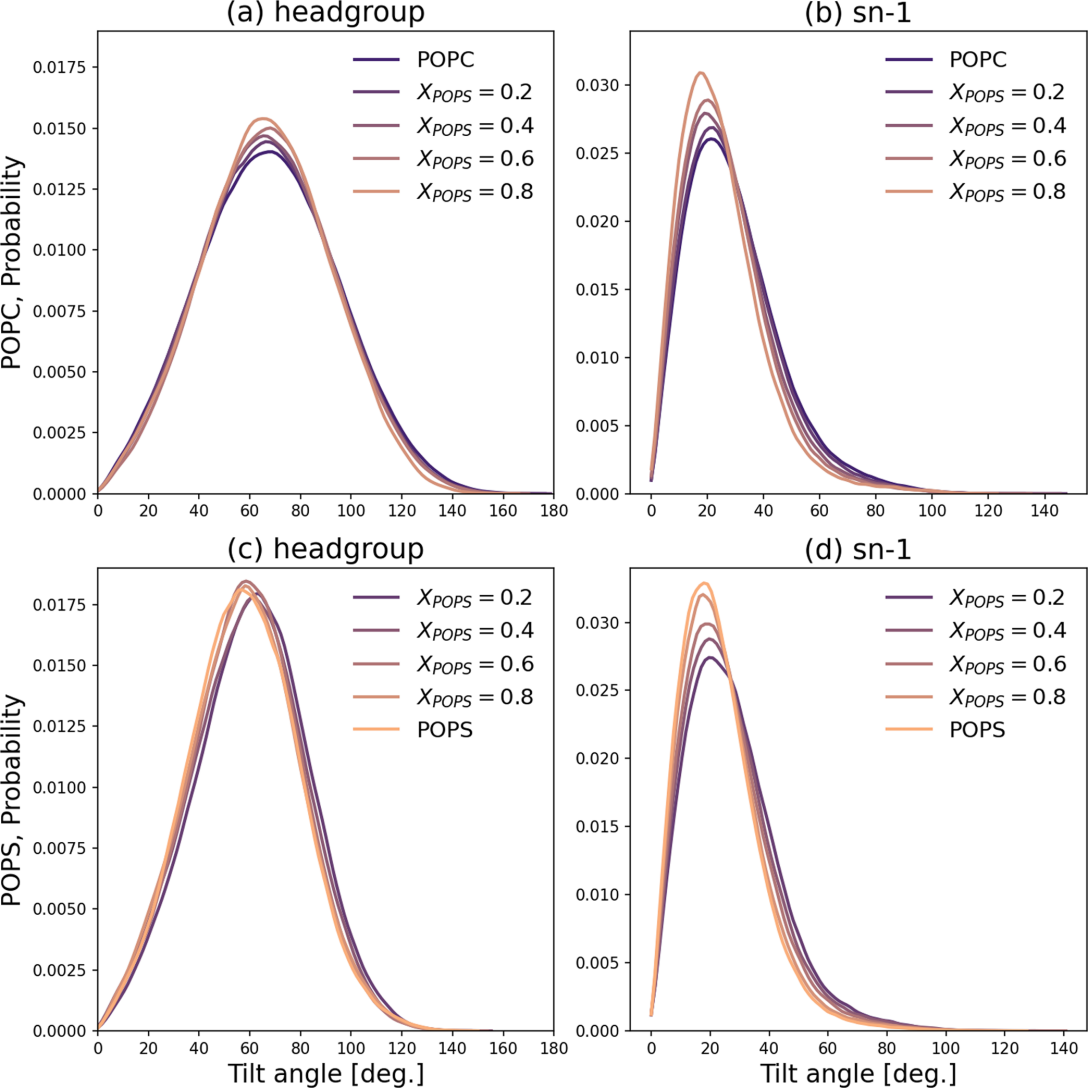
Probability distributions of the tilt angles: headgroup tilt angles θ_PC_ (a) and θ_PS_ (c) between the bilayer normal and the P–N (for POPC) and P–C (for POPS) vectors, respectively, and chain tilt angles θ_PCsn1_ (b) and θ_PSsn1_ (d) between the bilayer normal and the *sn*-1 chains of POPC and POPS, respectively. The probabilities were averaged over time and the number of repeats.

To analyze the lipid arrangement in the bilayer, we calculated four tilt angles (definitions are provided in Figure 1 and the MD Simulations section): headgroup tilt angles θ_PC_ and θ_PS_ (Figure 9a,c) between the bilayer normal and the P–N (for POPC) and P–C (for POPS) vectors, respectively, and chain tilt angles θ_PCsn1_ and θ_PSsn1_ (Figure 9b,d) between the bilayer normal and the *sn*-1 chains of POPC and POPS, respectively. On average, the POPC headgroup is tilted about 66° with respect to the membrane normal. In contrast, the POPS headgroups are less tilted (the maximum of the distribution is at ∼58°, as depicted in Figure 9c), with the average value of the θ_PS_ angle depending on the composition of the POPC/POPS bilayer. With an increase in the POPS content in the mixed membrane (Figure 9c), the θ_PS_ angle decreases (from ∼63° for *X*_POPS_ = 0.2 to ∼55° for the pure POPS bilayer). Interestingly, the addition of POPS to the POPC bilayer did not affect the θ_PC_ angle. For the acyl chain alignment, upon comparison of the pure systems, the angle θ_PCsn1_ (POPC chains) is larger than θ_PSsn1_ (POPS chains) by about 5°, suggesting less compact chain packing in the pure POPC bilayer. This correlation aligns with the fact that the APL of POPC and θ_PC_ is larger than that for POPS. The chain tilt angles for the mixed systems fall within the range between values for pure bilayers and are consistent with the lipid composition. The extent of POPC bilayer reorganization induced by the addition of POPS lipids appears to be consistent with the results of previous studies. For instance, Martinez-Seara et al.^53^ reported that changing the position of the double bond along the 18-carbon acyl chains causes a rearrangement of their tilt angle within approximately 4°. On the other hand, lipid headgroups have more freedom to reorganize under the influence of various factors. For example, Bilkova et al.^54^ observed changes in the tilt angle of phosphatidylinositol 4,5-bisphosphate (PI(4,5)P_2_) headgroups ranging from 37 to 65° upon the introduction of different counterions.

## Conclusions

We carried out MD simulations and Langmuir monolayer experiments for systems composed of the zwitterionic lipid POPC and the negatively charged lipid POPS, which we treated as a model of the outer leaflet of a plasma membrane with exposed PS. The experimental results showed that both lipids exhibit miscibility at the micrometer scale over the entire concentration range. Admittedly, the positive excess free energy of mixing prevailed for most mole fractions (indicating the expansion of the POPC/POPS films compared to the ideal mixing of both lipids). This means that mixing is thermodynamically unfavorable, yet the deviation from ideal mixing is modest, resulting in the absence of observable phase separation at the microscale using BAM microscopy.

Our MD simulations revealed that the experimentally observed expansion of POPC/POPS layers, relative to the ideal mixing of the two lipids, primarily stems from the conformational change of the POPS acyl chains in the mixed membranes. In the presence of POPC, the ordering of the hydrocarbon chains in POPS decreases significantly compared to that in the pure POPS membrane. This causes an increase in the surface area for the PS and leads to an overall membrane expansion. The increase in the APL for POPS is in part offset by the increased ordering of POPC in the mixed membrane. It is also worth noting that changes in the conformation of lipid molecules in mixed membranes result in changes in the commonly considered lipid packing parameter (the ratio of the cross-sectional area of the acyl chain to the headgroup regions). In other words, the packing parameter of a given lipid in a mixed membrane is different compared to that in a pure membrane of that lipid.

The key findings that emerge from our study hold significant implications: (i) The electrostatic attraction in POPC–POPC and POPS–POPS pairs significantly surpasses that within mixed pairs. In particular, the electrostatic attraction between POPS molecules exceeds that between POPC molecules by nearly twofold. As a result, both POPS and POPC tend to surround themselves with the same type of lipid. This led to lateral separation of the lipids and the formation of nanodomains rich in the negatively charged lipid. (ii) The phosphoserine headgroup exerts a compelling effect on increasing the ordering of lipid acyl chains compared to phosphocholine. This influence involves the elongation of the acyl chains attached to the glycerol, effectively augmenting the length of the POPS molecule compared to that of POPC. Consequently, this alteration triggers a slight protrusion of the PS group, causing it to emerge slightly above the zwitterionic membrane plane. Moreover, the PS group adopts an orientation characterized by a narrower angle to the membrane’s plane. In essence, it assumes a more compact alignment in comparison with its phosphocholine counterpart. The interplay of these factors collectively leads to an amplified exposure of the PS groups relative to the PC groups on the membrane’s surface. This heightened exposure bears profound biological implications, indicating the critical role of the phosphoserine headgroup.

The strategic placement of the phosphoserine headgroup and the formation of PS nanodomains contribute to a more pronounced exposure of the PS groups on the surface of the zwitterionic membrane, accentuating their accessibility and potential interactions with various membrane receptors and water-soluble proteins. This spatial arrangement enhances the recognition potential of interacting proteins, facilitating their capacity to interact more effectively with the PS groups residing on the membrane’s surface. Numerous proteins have been identified as sensors of the exposed phosphatidylserine on the surface of apoptotic cells. Among these proteins are growth-arrest-specific 6 (GAS6) and protein S, which subsequently act as ligands for TAM receptors expressed on the surface of phagocytes, thereby initiating the phagocytosis of apoptotic cells.^55−57^ While it is acknowledged that a complete molecular understanding of phosphatidylserine sensing remains an ongoing challenge, our structural insights into the unique organization of phosphatidylserines represent a significant step toward a deeper comprehension of vital biological processes. This study provides the foundation for further research into the functional implications of PS organization in biological processes such as signaling, apoptosis, and cancer, in which intricate molecular interactions play a central role in the functionality of cellular membranes.

## Supporting Information Available

Radial distribution functions (RDFs) and cumulative RDFs for POPC–POPS pairs in the mixed lipid bilayers with *X*_POPS_ = 0.2, 0.4, 0.6, and 0.8 and definition of the PS headgroup vector used in the tilt angle analysis and the discussion of alternative definitions (PDF)

## Dedication

We dedicate this paper to the loving memory of Dr. Tomasz Róg, our good colleague, who passed away on November 26, 2021. Dr. Róg applied molecular dynamics simulations to study systems of biological and medical interest.
