# Supplementary material for: Deciphering Lipid Arrangement in Phosphatidylserine/Phosphatidylcholine Mixed Membranes: Simulations and Experiments
Source: Langmuir. 2023 Dec 14;39(51):18995–9007. doi: 10.1021/acs.langmuir.3c03061 (PMC10753890; doi:10.1021/acs.langmuir.3c03061)
Supplement: Supplementary file 1 — la3c03061_si_001.pdf [file la3c03061_si_001.pdf]

Supporting Information for Deciphering Lipid Arrangement in Phosphatidylserine/Phosphatidylcholine Mixed Membranes: Simulations and Experiments

Agata Żak,[†,$] *Ksenia Korshunova,*[‡,$] *Natan Rajtar,*[†] *Waldemar Kulig,*[*‡] *and Mariusz Kepczynski*[*†]

[†] Faculty of Chemistry, Jagiellonian University, Gronostajowa 2, 30-387 Kraków, Poland

[‡] Department of Physics, University of Helsinki, P.O. Box 64, FI-00014 Helsinki, Finland

AUTHOR EMAIL ADDRESS:

waldemar.kulig@helsinki.fi (W.K.), kepczyns@chemia.uj.edu.pl (M.K.)

Table of Contents

- Definition of the PS headgroup vector used in the tilt angle analysis and the discussion of alternative definitions.

- Radial distribution functions (RDFs) and cumulative RDFs for POPC-POPS pairs in the mixed lipid bilayers with X_{POPS} = 0.2, 0.4, 0.6, and 0.8.

1. Definition of phosphatidylserine headgroup vector

For POPS, we used an arbitrary choice of the headgroup vector, defined by the P and C atoms (the P-C vector, Figure S1). It is important to highlight that the P-C vector chosen here is not the sole option, and alternatives exist, such as the P-N vector, which combines the P atom and the N atom of the amino group. We recalculated the tilt angle distributions for the PS headgroups using the P-N vector for two systems with X_{POPS} = 0.2 and 1.0 (**Figure S1**). The alternative definition of the vector introduces a uniform shift of about 20 degrees (panels a and b in **Figure S1**) to all distributions, while preserving the observed trends. This shift can be explained by examining both vectors (P-C vs. P-N, see panel c in **Figure S1**) in the POPS structure. Notably, it becomes evident that both vector definitions convey the same information and can be used interchangeably. However, in our opinion, the P-C vector, connecting the P atom and the C atom to which both charged groups (carboxyl and amine) are attached, seems to be more readable compared to the C-N vector, which clearly distinguishes the positively charged group.

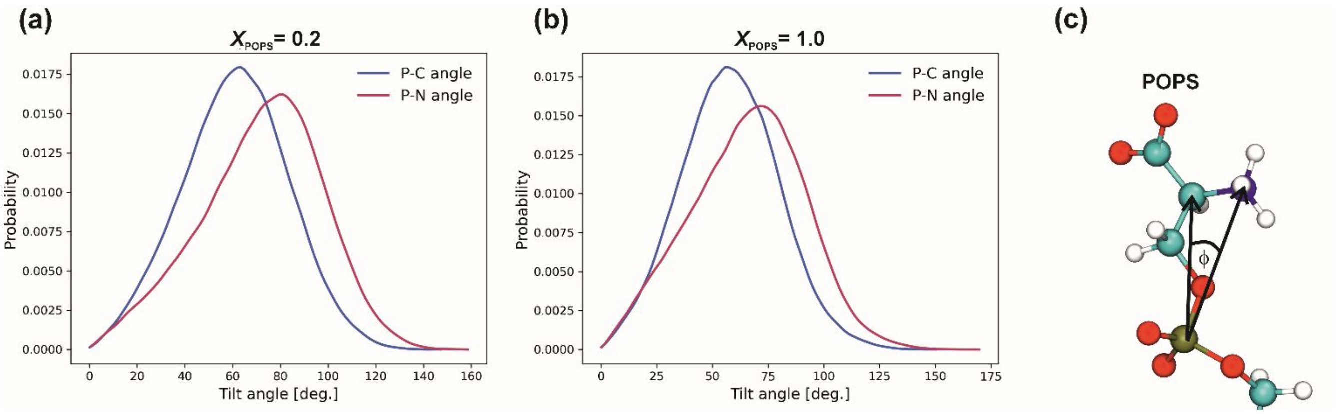

Figure S1. Probability distributions of tilt angles for the POPS headgroup in the systems with X_{POPS} = 0.2 (a) and 1.0 (b). Two distinct definitions of the headgroup tilt angle were used: the tilt angle between the bilayer normal and the P-N vector (red curve) and the tilt angle between the bilayer normal and the P-C vector (blue curve). Probabilities were averaged over time and the number of repeats. (c) A comparison between the definitions of P-C and P-N vectors.

2. **Radial distribution functions**

To further corroborate the contact analysis presented in the main manuscript and remove the cut-off dependency needed in the number of contacts analysis, we calculated the radial distribution functions (RDFs) and cumulative RDFs for POPC-POPS pairs in the mixed lipid bilayers with X_{POPS} = 0.2, 0.4, 0.6, and 0.8. These are presented in **Figure S2**. To aid the comparison between the cumulative RDFs and the number of contacts analysis, vertical dotted lines were added (see panel (b) in **Figure S2**), showing the cut-off distance used in the contact analysis.

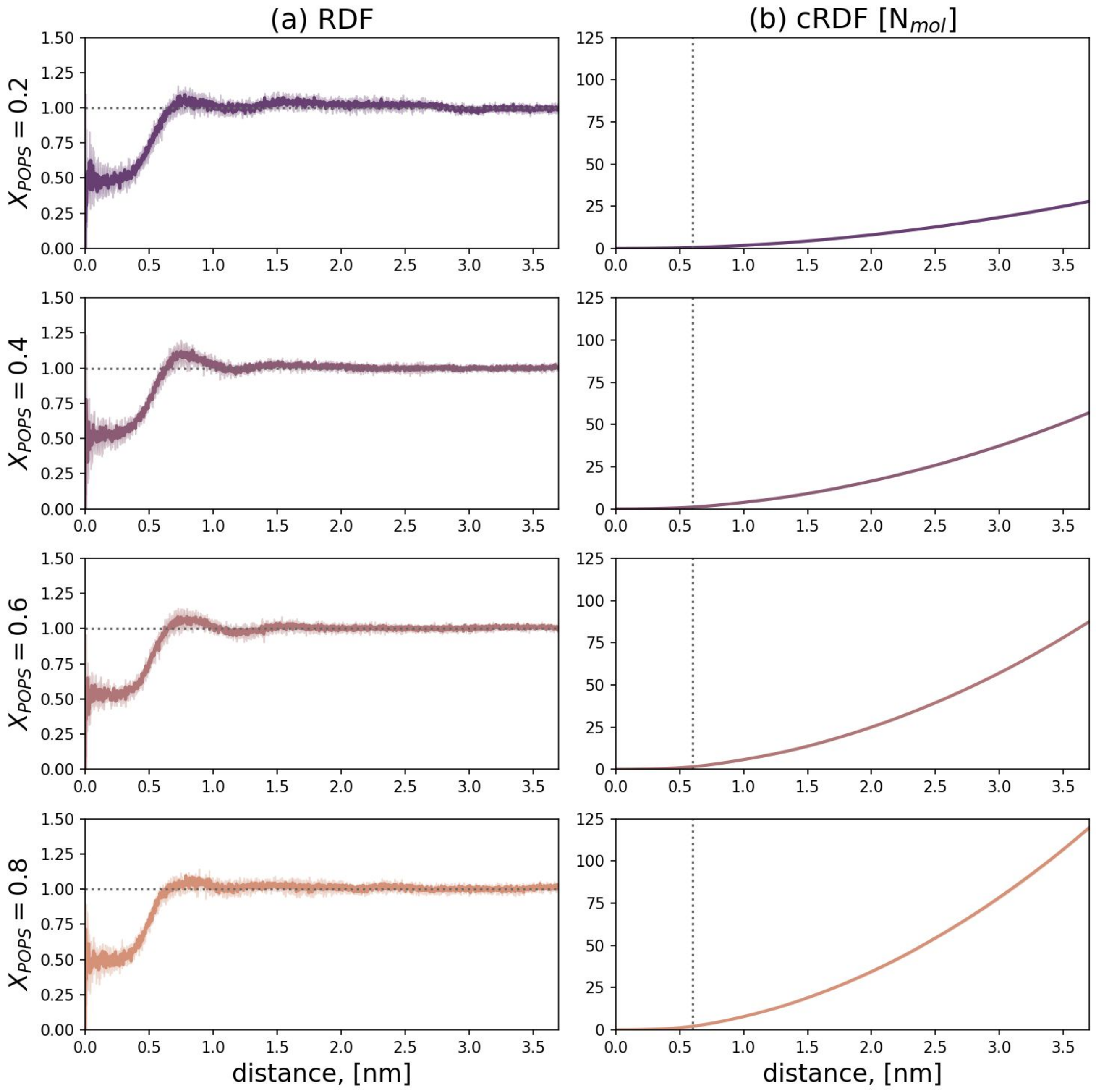

Figure S2. (a) Radial distribution functions (RDFs) for POPC-POPS pairs in the mixed lipid bilayers with X_{POPS} = 0.2, 0.4, 0.6, and 0.8. RDFs are calculated as an average over simulation repeats (dark colors) with the error bars (bright colors) evaluated as standard deviation of repeats. **(b)** Cumulative RDFs for POPC-POPS pairs in the mixed lipid bilayers with X_{POPS} = 0.2, 0.4, 0.6, and 0.8. The dotted vertical line corresponds to the distance of 0.6 nm (the cut-off used in the contact analysis reported in main manuscript).
